# Predictors and Risk Assessment Models for Venous Thromboembolism in Patients Diagnosed with Lymphoma: A Systematic Review

**DOI:** 10.3390/curroncol33070401

**Published:** 2026-07-04

**Authors:** Anca Maria Pop, Markus Rütti

**Affiliations:** 1 Department of Internal Medicine, HOCH Health Ostschweiz, Hospital Wil, 9007 St. Gallen, Switzerland; 2 Department of Medical Oncology and Hematology, HOCH Health Ostschweiz, Cantonal Hospital St. Gallen, 9007 St. Gallen, Switzerland

**Keywords:** lymphoma, venous thromboembolism, risk assessment model, predictors, thrombosis

## Abstract

Among hematological malignancies, lymphoma is associated with an increased incidence of venous thromboembolism (VTE) ranging between 4 and 12%. Although Khorana score was validated for stratifying VTE risk in cancer, its discrimination reliability in lymphoma is reduced by the lack of specific predictors. The aim of this systematic review was to summarize the evidence regarding predictors and available risk assessment models (RAMs) for VTE in patients with lymphoma. A systematic search was conducted on PubMed, Embase and Scopus in order to identify papers published until February 2026, which evaluated predictors and RAMs for VTE in patients diagnosed with lymphoma. Out of 592 evaluated papers, 44 met the inclusion criteria. The widely used Khorana score failed to appropriately identify patients with lymphoma at high risk for VTE, while the Thrombosis Lymphoma predictive score (ThroLy) showed modest improvement. Strong predictors for VTE were a poor performance status, older age, previous history of VTE, the use of central venous catheters, and bulky disease. However, the lack of external validation, the small sample size and bias due to confounding factors limit the generalizability of the results. Therefore, larger studies with external validation cohorts are needed to design lymphoma-specific RAMs and to identify predictors with high discrimination power.

## Simple Summary

Lymphoma is the most common hematological malignancy and is associated with an increased risk for venous thromboembolism (VTE), especially in patients with aggressive non-Hodgkin lymphoma where VTE incidence approaches 12%. Although current guidelines recommend the use of prophylactic anticoagulation in patients with high risk for VTE, the existing evidence regarding the appropriate risk stratification in patients with lymphoma is still insufficient. We aimed to critically analyze the existing evidence regarding predictors and new developed risk assessment models for VTE in patients diagnosed with lymphoma and to evaluate the already existing models for VTE risk stratification in cancer patients. The role of the Khorana score in identifying patients with lymphoma at high risk for VTE has been under intense scrutiny, alongside the evaluation of more recently developed assessment models, which currently show significant risk of bias.

## 1. Introduction

**Figure 1:**
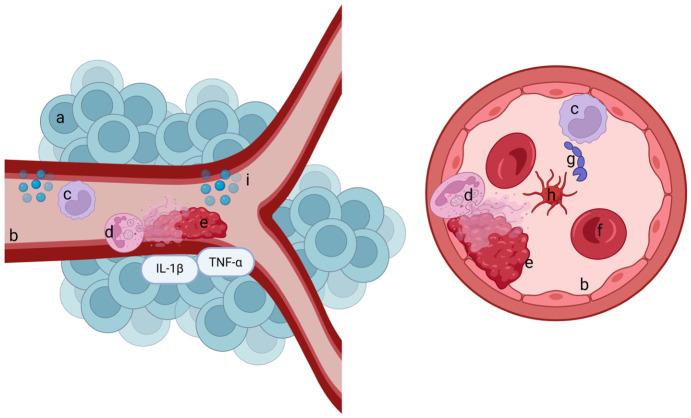
Schematic illustration of the pathophysiological mechanisms leading to venous thrombosis in lymphoma. The clonal lymphoma cells release various cytokines, among them TNF-α and IL-1β, which enhance the endothelial activation. The circulating CD14-monocytes release tissue factor (TF), which leads to platelet degranulation and adhesion. Moreover, the platelet adhesion is favored by the activated neutrophils, which build neutrophil extracellular traps (NETs). a = clonal lymphoma cells, b = blood vessel in longitudinal and transversal section, c = activated CD14-monocyte containing tissue factor, d = neutrophil with adjacent neutrophil extracellular traps, e = thrombus, f = circulating erythrocyte, g = activated tissue factor, h = degranulated platelet, i = various cytokines released by lymphoma cells. Created in BioRender, https://BioRender.com/9y5mlmc (accessed on 30 June 2026).

Venous thromboembolism (VTE) is a frequent and serious complication in patients with cancer, being the second cause of mortality after cancer progression itself [1]. Among hematological malignancies, lymphoma is associated with an increased risk for VTE [2]; data from the literature report a VTE prevalence of approximately 4% in Hodgkin lymphoma (HL), which can increase up to 12% in non-Hodgkin lymphoma (NHL), especially in diffuse large B-cell lymphoma (DLBCL) [3]. The VTE risk is particularly high in the first months after diagnosis and is enhanced through several pathophysiological mechanisms [4]. Patients with lymphoma usually exhibit an increased inflammatory and hypercoagulant activity, which also worsens disease prognosis. The high level of proinflammatory cytokines leads to the activation of the coagulation cascade through the secretion of tissue factor by circulating blood monocytes, which further activates the extrinsic coagulation pathway. Moreover, thrombosis involves a supplementary inflammatory response in the vessel wall, facilitating the expression of adhesion molecules and the activation of neutrophils [5]. A unique feature of lymphoma is the complex inflammatory milieu leading to immune dysregulation and lastly to lymphomagenesis. Various polymorphisms of interleukin (IL)-6, IL-10, as well as high levels of tumor necrosis factor alpha (TNF-α) are associated with NHL and are also involved in thrombogenesis [6] (Figure 1).

The current guidelines of the European Society for Medical Oncology recommend the use of prophylactic anticoagulation in patients with hematological malignancies with high risk for VTE [7]; however, the simultaneously increased hemorrhagic risk in this category of malignancies and the chemotherapy-associated thrombocytopenia limit the wide use of prophylactic anticoagulation [8,9]. VTE prophylaxis is widely prescribed for cancer patients admitted to hospitals, but evidence regarding the appropriate thromboprophylaxis for ambulatory settings is still insufficient [7]. Several risk assessment models (RAMs) for VTE in patients with cancer were developed, with the most widely known being the Khorana score [10]. However, the Khorana score addresses cancer patients as a whole entity, without taking lymphoma-specific particularities into consideration. In an effort to better define precise RAMs for VTE in lymphoma patients, various multivariable models and predictors were evaluated in the last decade; however, most of them did not reach clinical significance.

The aim of this systematic review was to summarize the available evidence regarding already-developed RAMs and predictors for VTE in patients diagnosed with lymphoma.

## 2. Materials and Methods

### 2.1. Algorithm for the Systematic Search and Selection of Studies

A systematic literature search was performed according to the Preferred Reporting Items for Systematic Reviews and Meta-analysis (PRISMA) guidelines [11]. The research protocol was registered and approved by the International Prospective Register for Systematic Reviews and Meta-analysis (PROSPERO) under the number CRD420251237060. The systematic database search was performed on PubMed, Scopus and Embase using combinations of the keywords “venous thromboembolism”, “pulmonary embolism”, “deep vein thrombosis” and “lymphoma”, which were aligned with MeSH and Emtree vocabulary. The detailed search strategy and the PRISMA checklist are included in the Supplementary Files S1 and S2. All available original research articles focusing on RAMs and predictors of VTE in patients diagnosed with lymphoma, published from inception until February 2026 (last access of all databases on 7 March 2026), have been further screened based on eligibility criteria. Duplicates were screened and appropriately verified using Mendeley.

The inclusion criteria were: prospective and retrospective studies written in English with a minimum level of evidence of IV, and studies evaluating patients over 18 years of age and with a histological diagnosis of HL or NHL under treatment, either ambulatory or hospitalized. The type of the discrimination statistics reported or the validation methods used in the studies were not predefined outcomes. The exclusion criteria were: studies also focusing on other hematological malignancies other than lymphoma, studies including pediatric population, case reports/series and reviews and non-English articles. The titles and abstracts of the selected papers were evaluated independently by both authors. In case of disagreement, the final decision of inclusion was met upon consensus.

### 2.2. Quality Assessment

The quality of the studied prediction models was evaluated based on the PROBAST tool (Prediction model Risk of Bias Assessment Tool), which classifies prediction models as having low, high or unclear risk of bias by assessing four domains: participants, predictors, outcome and analysis [12]. Since the evaluation of one domain as having high risk of bias leads to evaluation of the whole prediction model as having high risk of bias, many papers fail to achieve a consistent quality based on the PROBAST tool. However, for descriptive purposes, all papers focusing on prediction models were included in the final analysis with the necessary explanations regarding bias concern. Studies assessing only predictors for VTE were evaluated based on the QUIPS tool (Quality In Prognosis Studies), which evaluates six domains including participation, attrition, prognostic factor and outcome measurement, confounding and statistical analysis [13]. The studies can be classified as having low (maximum one criterion evaluated as having moderate risk of bias), moderate (four criteria with low risk of bias and two with moderate risk of bias) or high risk of bias (maximum three criteria with low risk and at least three criteria with moderate risk of bias or one criterion with high risk of bias).

### 2.3. Data Extraction and Statistical Analysis

Data from the selected studies were extracted by the first author (A.M.P.), who summarized the baseline characteristics of the studies, such as author names, study design, year of publication, number of participants, histological type of lymphoma, prior use of thromboprophylaxis and the type of predictors and RAMs evaluated. Supplementary Information on the outcome reported in the study in the form of area under the curve (AUC), C-index, sensitivity and specificity, odds (OR), and hazard ratio (HR) was also extracted by the first author into a Microsoft Excel spreadsheet (MS Microsoft, Redmond, WA, USA). After the process of extraction, all collected data were reviewed by the senior author (M.R.) and any disagreements were resolved upon discussion among the two authors. Continuous variables were presented as means with confidence intervals. A value of *p* < 0.05 was considered statistically significant.

## 3. Results

**Figure 2:**
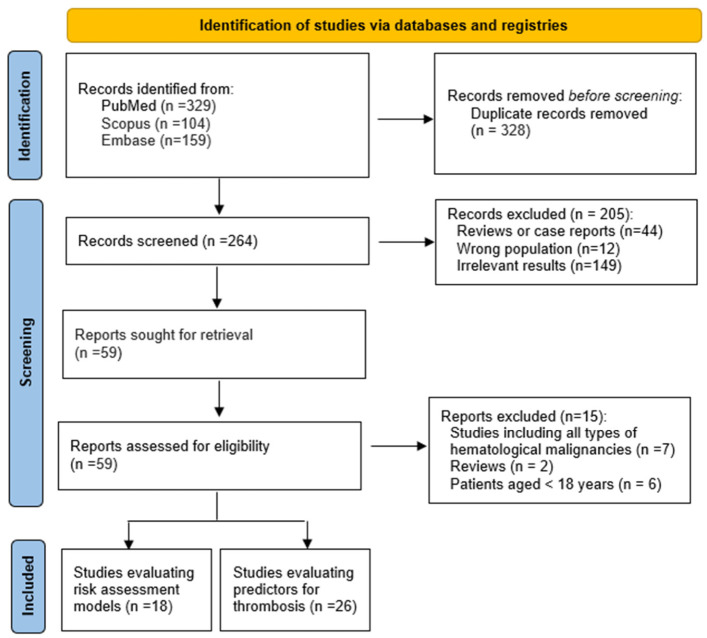
PRISMA diagram illustrating study selection protocol.

The initial literature search identified 592 papers, out of which 44 met the inclusion criteria according to the selection protocol (Figure 2). The results were evaluated separately for studies focusing on multivariable RAMs and for studies assessing VTE predictors without including them in a RAM.

### 3.1. Studies Evaluating RAMs

Out of the studies evaluating RAMs, thirteen were single-center [14,15,16,17,18,19,20,21,22,23,24,25,26] and mainly conducted in Asia [14,15,18,19,20,21,22,23,26,27,28]. The prevalence of lymphoma ranged between 4 and 15%, depending on the histological type of lymphoma predominantly included. Abdel et al. [14,15] conducted two studies focusing exclusively on DLBCL and reported prevalences of VTE between 13.5 and 15%. Within a follow-up of 6 months, He et al. [19] found a VTE prevalence of 10.1% among hospitalized patients with NHL. By including all types of lymphoma, Liang et al. [21] reported a VTE prevalence of 4.9%, while Jiang et al. [27] recorded a prevalence of 10.8% in hospitalized patients.

With regard to the nomograms proposed for VTE prediction, the included studies focused either on evaluating or repurposing already existing scores or on developing new VTE risk assessment models. Abdel [14] and Hantrakun [18] focused on the repurposing of the already existing IPI index. Antic et al. [29] developed the Thrombosis–Lymphoma (ThroLy) predictive score, which was further applied in five studies, considered external validation studies [15,16,20,22,25]. The already existing Khorana score for VTE prediction in cancer was employed in six studies [16,17,20,22,24,30], while ten studies focused on developing new nomograms or prediction models for VTE risk in patients with lymphoma [16,17,19,21,23,26,27,28,29,31].

The risk of bias was high in the majority of the included papers. Only three papers reported competing risks such as death [18,30,31]. Moreover, among the newly proposed prediction models, only three received external validation [27,28,31], which is a crucial criterion for the analysis domain of the PROBAST tool. The detailed description of the risk of bias among the included studies is presented in Table 1, and the characteristics of the evaluated studies are summarized in Table 2.

The most frequently evaluated predictors, identified by multivariate analysis, are summarized in Table 3.

#### 3.1.1. Khorana Score

The Khorana score showed an insufficient discrimination power in lymphoma patients, mainly due to the lack of lymphoma-specific predictors. Rupa et al. [24] recorded a positive predictive value of 15%, a negative predictive value of 82% and a C-statistic of 0.51 corresponding to a near-chance discrimination, in the context of an appropriate sample size and a rate of events per variable up to 12. Moreover, Rupa et al. [24] found the histological type of DLBCL, bulky disease and poor prognosis to be associated with VTE risk; however, they did not include these parameters in a new VTE RAM. These results are in accordance with the findings of other studies, which reported an area under the curve of the Khorana score ranging between 0.502 and 0.639 [16,20,23,26]. In a smaller study sample with fewer events per variable, Ma’koseh et al. [22] also found no correlation between the occurrence of VTE and a Khorana score > 2. However, Santi et al. [30] concluded that a higher Khorana score is statistically significantly positively associated with the VTE risk, but they did not report any discrimination statistics, which makes the difference between discrimination power and pure statistical association difficult.

#### 3.1.2. Thrombosis Lymphoma (ThroLy) Predictive Score

The ThroLy score was developed by Antic et al. [29] and recorded a C-statistic of 0.85–0.87 in the derivation and validation cohort. However, the authors conducted no external validation and also included the occurrence of arterial thrombosis, which exhibits a different pathophysiological background compared to VTE. The score recorded a high positive predictive value of 65.2% in high-risk categories (≥4), but with a wide confidence interval ranging from 42 to 83%. Other studies acting as external validation cohorts for the ThroLy score [16,20,23] reported areas under the curve of the ThroLy score ranging from 0.579 to 0.695. Rupa-Matysek et al. [25] found that 48% of the VTE events occur in low-risk ThroLy categories and calculated a C-statistic of 0.55, which was modestly better than the C-statistic of 0.51 recorded by the Khorana score in the same cohort of lymphoma patients.

#### 3.1.3. International Prognostic Index

Abdel et al. [14] used the already available International Prognostic Index for non-Hodgkin lymphoma and found a 19.8% VTE occurrence in patients with high- and high–intermediate scores compared to 9.7% in those with low- and low–intermediate scores; however, they did not report any discrimination statistics. Hantrakun et al. [18] obtained a C-statistic of 0.65 based on the age-adjusted IPI in a cohort of 591 patients, but the low rate of events per variable might increase the risk of overfitting.

#### 3.1.4. Newly Developed VTE Risk Assessment Models

Ma et al. [31] developed a VTE RAM with seven routinely accessible predictors selected using LASSO regression. The study population included 13,025 patients, divided into one training and two external validation cohorts. The authors obtained a C-statistic of 0.68–0.72 in all sets of patients, with narrow confidence intervals and based on the highest rate of events per variable among available studies, which strengthened the generalizability of the results and decreased the risk of bias.

Dharmavaram et al. [17] added four lymphoma-relevant parameters to the Khorana score (bulky disease, lymphoma histological subtype, albumin and leukocyte count), which increased the C-index at 2 years follow-up from 0.601, as obtained for the Khorana score, to 0.775 for the proposed new model.

Liang et al. [21] developed a nomogram for VTE prediction, including risk factors of age, gender, D-dimer level, platelet count, and the chemotherapy cycle, and obtained an area under the curve of 0.838 with a high Royston D statistic and acceptable rate of events per variable, indicating a robust internal validation and good discrimination power.

Pan et al. [23] designed a similar nomogram, taking into consideration already validated risk factors for VTE such as ECOG performance score, prior VTE history, the use of central venous catheters, D-dimer levels, and the presence of coronary heart disease, and obtained an area under the curve at 1 year of 0.818, which was higher than the values of 0.587 and 0.527 recorded for the Khorana and ThroLy scores, respectively; however, at 2 years, the area under the curve decreased to 0.733, which indicated a lower power of discrimination over time.

Yang et al. [26] reported an area under the curve of 0.731 for a five-variable nomogram, which was also higher than the value of 0.557 recorded for the Khorana score in the same cohort of patients.

A distinctive study is the one conducted by Wang et al. [28], in which the authors focused exclusively on the PICC-associated thrombosis; based on five variables, the authors obtained a very high area under the curve of 0.907 in the training set and of 0.896 in the external validation set, respectively; however, the wide confidence intervals and the low rate of events per variable increased the risk of overfitting.

### 3.2. Studies Evaluating VTE Predictors

**Figure 3:**
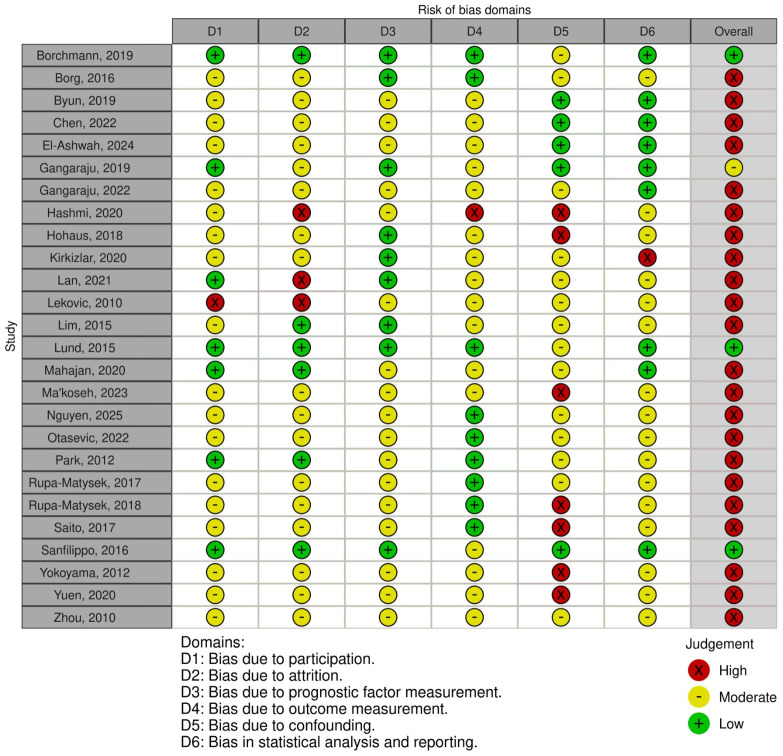
QUIPS risk of bias assessment of the included studies [2,4,6,32,33,34,35,36,37,38,39,40,41,42,43,44,45,46,47,48,49,50,51,52,53,54]. Created in *robvis* [55].

Twenty-six studies evaluating predictors for VTE were included in the final analysis [2,4,6,32,33,34,35,36,37,38,39,40,41,42,43,44,45,46,47,48,49,50,51,52,53,54]. The detailed description of the risk of bias among the included studies is presented in Figure 3.

The risk of bias was considered high in the majority of studies, especially due to bias in considering the confounding factors. The prevalence of VTE ranged between 3 and 35%, depending on the histological type of lymphoma included. Higher age was associated with two- to threefold higher odds of developing VTE [34,35,42,47], while a poor performance status was also associated with higher VTE risk [33,34,38,39,42,52]. Previous VTE history was a strong predictor for thrombosis development, associated with four- to fivefold higher odds of VTE development [33,44,50,51]. Borg et al. [33] reported a VTE prevalence of 11% in 289 patients with DLBCL, although 100 patients were already receiving thromboprophylaxis; the authors identified the past history of VTE as the strongest predictor for a new thrombotic event. In two large cohorts of patients with lymphoma, Borchmann [32] and Lund [29] obtained a VTE prevalence of only 3%, significantly associated with the use of central venous catheters (CVC). Moreover, in a large cohort of patients with DLBCL and FL, Sanfilippo et al. [51] reported significantly higher odds of VTE during chemotherapy and also in patients with previous VTE.

The characteristics of the included studies with evaluated predictors for VTE are summarized in Table 4.

## 4. Discussion

Patients with lymphoma are at high risk for VTE due to the active disease itself, but also due to chemotherapy [15], insertion of CVCs, or hospitalizations leading to immobilization [45,56]. In a large cohort study conducted by Martens et al. [57], aggressive NHL was associated with the highest VTE incidence among hematological malignancies, second to acute lymphoblastic leukemia.

The Khorana score remains the current standard for prescribing prophylactic anticoagulation in patients with cancer, showing reasonable performance in solid tumors; however, its ability to discriminate patients with lymphoma who are at high risk for VTE is insufficient, since all histological types of lymphoma are categorized as one entity, ranging from indolent to very aggressive forms [29]. Our systematic review identified 18 studies focusing on multivariable RAMs and 26 studies evaluating predictors for VTE in lymphoma patients. Most of them were single-center studies that lacked external validation, making generalization of the obtained results rather difficult. Furthermore, in most studies, VTE was defined as DVT of either the upper or the lower extremity, which are separate entities with different underlying pathophysiological mechanisms: DVT of the upper extremity is CVC-related, while DVT of the lower extremity is a result of the inflammatory milieu and may lead to PE [57].

Lymphoma-specific RAMs for VTE frequently used predictors such as the ECOG performance status, extranodal localization, prior history of VTE and the histological subtype of lymphoma, which were previously identified in prevalence studies. The poor performance status in lymphoma patients is associated with lower overall and progression-free survival [39]. Moreover, available data in the literature identify a poor performance status, defined as ECOG ≥ 2, to be correlated with a higher VTE risk [52,58]. Nguyen et al. [46] found that patients with newly diagnosed lymphoma have fivefold higher odds of developing VTE compared to those with ECOG 0–1. In the same manner, patients with prolonged immobilization are at increased risk of developing VTE, especially in cases of primary central nervous system lymphoma (PCNSL), where VTE prevalence may increase up to almost 60% [59] or in frail patients with DLBCL [60]. Bulky disease was also identified as a risk factor for VTE development [46], mainly due to the compression effect and venous stasis caused by large mediastinal masses [61]. Moreover, older age associated with poorer health condition in patients diagnosed with lymphoma may further augment the VTE risk [47].

The thrombosis risk in patients with lymphoma is considered high at the beginning of chemotherapy, leading to possible hospitalizations due to VTE [62,63]. Liang et al. [21] found that the VTE risk is increased, especially during the 6th to 10th or after the 11th chemotherapy cycle, explaining that the cumulative effect of chemotherapy induces the damage of vascular cells, platelet activation and release of procoagulant proteins [21,64]. Li et al. [65] found a higher VTE incidence in female patients, in those with platelet count abnormality and Ann Arbor stage III/IV undergoing chemotherapy. On the other hand, Borg et al. [33] concluded that the VTE risk is especially high before treatment initiation and it further decreases during and after chemotherapy. Among the most widely used chemotherapeutic agents, doxorubicin is associated with a three- to fourfold increase in the VTE risk [51,54,66,67]. Moreover, asparaginase derivatives, which are used in lymphoblastic lymphoma treated with acute lymphoblastic leukemia protocols, carry a well-known, very high risk for VTE development, especially due to the decreased synthesis of anticoagulant proteins [68,69]. Novel therapies, such as chimeric antigen receptor (CAR) T-cell therapy used for refractory or relapsed lymphomas, are also associated with increased VTE incidence due to the advanced disease itself and the systemic inflammation caused by therapy [38].

The histological variety is a unique feature of lymphoma, which is associated with disease aggressiveness and therefore with VTE risk. NHL is three to five times more frequent than HL [70] and among NHL types, DLBCL is the most common and aggressive form, accounting for up to 30–40% of cases [71]. Gangaraju et al. [37] found an eightfold higher risk of VTE in elderly patients with DLBCL compared to healthy controls. In a large cohort of patients from Denmark, Lund et al. [43] found the highest VTE incidence at one year after diagnosis in patients with peripheral T-cell lymphoma (4.3%) and DLBCL (4.2%). In a meta-analysis comprising over 18.000 patients, Caruso et al. [72] reported a statistically significant higher VTE incidence in NHL (6.5%) compared to HL (4.7%).

Our review aimed to characterize the efficiency of novel and already existing RAMs for VTE detection in patients diagnosed with lymphoma, starting from the premise that general RAMs for VTE like Khorana score lack appropriate discrimination ability in lymphoma. Moreover, we aimed to summarize the available evidence on the studied predictors for VTE detection in patients diagnosed with lymphoma. Ten out of the 18 included RAMs were newly developed, while six were designed as validation cohorts for the Khorana and ThroLy scores. The most frequent histological type of lymphoma was DLBCL, which is associated with a high VTE risk and may consequently lead to the overestimation of the VTE burden in patients with lymphoma. Moreover, eleven studies originated from Asia, where the incidence of NHL is constantly increasing [73], thus limiting the generalizability of the results. Most studies evaluating RAMs excluded patients who were receiving anticoagulants, as previous thromboprophylaxis represents an important confounding factor. However, there are still many studies evaluating predictors, where there were no detailed data regarding previous anticoagulation or where patients already receiving thromboprophylaxis developed VTE [32,33,45,52,53].

The generalizability of the results is mainly limited due to the significant risk of bias, consisting in the single-center design with insufficient sample size, lack of external validation and discrimination statistics, as well as the heterogeneous VTE definition (inclusion of catheter-associated VTE). The identified predictors were mainly selected using univariate analysis, which is a common practice with an increased risk of overfitting, especially when not using cross-validation or independent training sets for the selection of predictors [74]. Ma et al. [31] developed a promising and robust RAM for VTE, externally validated in two cohorts and with reliable predictors; the model also differentiates between overall VTE and lower extremity DVT/PE, emphasizing once again the different pathophysiological mechanisms underlying these conditions. The selected predictors are similar within the selected studies and comprise the ECOG performance status, Ann Arbor stage, histological type of lymphoma, D-dimer and hemoglobin levels, and extranodal and bulky disease.

A critical issue in the design of clinical prediction models is the number of events per variable (EPV). Historically, a rule of thumb of ≥10 EPV should be sufficient for achieving statistical significance; however, in clinical practice, the risk of overfitting or even of paradoxical associations is increased when the number of EPV is low [75]. Ma et al. [31] achieved a number of over 70 EPV for a RAM evaluating seven covariates, which, according to Balachandran et al. [76], meets the requirements for nomograms in oncology. However, the authors report the results as C-statistic, which assesses exclusively discrimination capacity, without weighing clinical benefit. In order to translate into clinical significance, decision curve analysis should be employed in order to evaluate clinical utility beyond simple discrimination [77].

### 4.1. Limitations and Future Directions

An important limitation of our systematic review is the lack of a meta-analysis, which is mainly driven by the substantial data heterogeneity. The relevant variability in lymphoma subtypes with different associated VTE risks (for example DLBCL versus HL) as well as the treatment settings limit pooling and comparability. Moreover, most of the studies present retrospective data and do not differentiate patients according to histological subtype of lymphoma or type of thrombosis (upper or lower extremity). Another important aspect is the lack of external validation in most of the reviewed models, which together with the low number of EPV weaken the strength and generalizability of the identified predictors for VTE in patients with lymphoma. We acknowledge that a closer look is needed when analyzing the way in which the noted bias and confounding variables affect the predictor validity and hope that this review may aid in the design of future studies with strengthened methodology.

### 4.2. Clinical Implications

Thromboprophylaxis is recommended by guidelines in hospitalized patients with hematological malignancies [78]. However, there is no clear consensus regarding thromboprophylaxis in ambulatory settings, since most of the available data regarding the efficiency and safety of anticoagulant agents originate from studies evaluating the treatment, instead of the prophylaxis of VTE. Direct oral anticoagulants exhibited an acceptable safety profile compared to the standard low-molecular-weight heparin and may be considered in stable patients with lymphoma. Due to the existing evidence gaps, a tailored approach, which takes into account the histological type of lymphoma (especially PCNSL and DLBCL), previous history of VTE, performance status, age, the use of CVCs, and associated risks, for example thrombocytopenia, is required in order to guide the decision of ambulatory thromboprophylaxis.

## 5. Conclusions

VTE is a frequent and serious complication in patients with lymphoma. Although robust predictors for VTE risk stratification were defined (ECOG performance status, VTE history, histological subtype), the substantial risk of bias in most of the studies, the retrospective and heterogeneous nature of data, the unclear handling of confounders, and lack of external validation make the evidence regarding an appropriate VTE prediction tool insufficient. Larger, multicentric studies with external validation cohorts are required in order to design VTE RAMs for patients with lymphoma.

## Figures and Tables

**Table 1:** PROBAST risk of bias assessment of the included studies.

Author, Year	Risk of Bias				Applicability			Overall	
	1. Participants	2. Predictors	3. Outcome	4. Analysis	1. Participants	2. Predictors	3. Outcome	Risk of Bias	Applicability
Abdel, 2021 [14]	+	+	+	-	-	+	+	-	-
Abdel, 2021 [15]	+	+	?	-	?	+	?	-	?
Antic, 2016 [29]	+	+	?	?	+	+	?	?	?
Bastos-Oreiro, 2021 [16]	-	-	?	-	-	-	?	-	-
Dharmavaram, 2020 [17]	?	+	?	?	?	+	?	?	?
Hantrakun, 2021 [18]	+	+	+	?	?	+	+	?	?
He, 2025 [19]	-	-	?	-	-	-	?	-	-
Jiang, 2025 [27]	+	+	+	-	+	?	+	-	?
Li, 2024 [20]	?	-	?	-	?	-	?	-	-
Liang, 2023 [21]	+	+	+	+	?	+	+	+	?
Ma, 2024 [31]	+	+	+	+	+	+	+	+	+
Ma’koseh, 2024 [22]	-	+	?	-	?	+	?	-	?
Pan, 2015 [23]	+	+	?	-	?	+	?	-	?
Rupa-Matysek, 2018 [24]	?	+	+	?	?	+	+	?	?
Rupa-Matysek, 2018 [25]	?	+	+	?	?	+	+	?	?
Santi, 2017 [30]	+	+	+	?	+	+	+	?	+
Wang, 2024 [28]	?	?	+	-	?	?	+	-	?
Yang, 2021 [26]	+	?	?	-	?	-	?	-	-
*Explanation*	*Low risk of bias (+)*			*Unclear risk of bias (?)*			*High risk of bias (-)*		

**Table 2:** Characteristics of the studies assessing RAMs for VTE prediction.

Authors, Year, Type of Study	Number of Patients	Type of Lymphoma	Studied Model	Type of Validation	Studied Variables	VTE Incidence and Events per Variable	Thromboprophylaxis/Thrombophilia Screening	Results	Risk of Bias
Abdel, 2021, Retrospective [14]	373	DLBCL	Lymphoma International Prognostic Index	N/A	ECOG, LDH, age, stage, extranodal disease	56 patients (15%): 15 DVT in lower extremities 15 DVT in upper extremities 15 PE 5 DVT and PE 6 others EPV: ~10	No information on prior thromboprophylaxis or thrombophilia screening	High VTE rates in patients with: Poor performance status (ECOG 2–4: 26.2% vs. ECOG 0–1: 12.9%) High LDH levels (19% vs. 9.4%) Based on age-adjusted IPI: VTE in 9.7% patients with “low and low–intermediate” scores VTE in 19.8% patients with “high and high–intermediate” scores (*p* = 0.02)	*Strengths:* Validated tool *Limitations:* Single center and small sample size No discrimination statistics
Abdel, 2021, Retrospective [15]	524	DLBCL	ThroLy	N/A	Previous VTE/AMI/stroke, ECOG 2–4, BMI > 30 kg/m^2^, extranodal localization, mediastinal involvement, neutropenia during therapy, hemoglobin < 100 g/L	71 patients (13.5%): 26 (37%) DVT of lower extremities 20 (28.2%) DVT of upper extremities 22 (31%) isolated PE EPV: ~10	No thromboprophylaxis in ambulatory patients. No information on thrombophilia screening.	VTE developed in 44 (17.2%) high-risk patients (*n* = 256) compared to 27 (10.1%) in the low-risk group (*n* = 268), *p* = 0.038 Risk factors for VTE: Hemoglobin < 100 g/L (OR 2.79, 95% CI 1.42–5.49) Bulky disease (OR 2.22, 95% CI 1.31–3.75)	*Strengths:* Standardized score *Limitations*: Single center and small sample size No discrimination statistics
Antic, 2016, Retrospective [29]	1820	NHL, HL, CLL/SLL	ThroLy	Internal	Previous VTE/AMI/stroke, ECOG 2–4, BMI > 30 kg/m^2^, extranodal localization, mediastinal involvement, neutropenia during therapy, hemoglobin < 100 g/L	74 patients (4.06%): 37 DVT in extremities 16 internal jugular veins 5 isolated PE 14 superficial vein thrombosis Thrombosis in other organs. EPV: ~10	3% of patients received thromboprophylaxis at baseline. No information on thrombophilia screening.	*At risk scores (>1):* NPV 98.5% (97.5–99.1%) PPV 25.1% (19.2–31.8%) Sensitivity 75.4% (63.1–85.2%) Specificity 87.5% (85.5–89.4%) High-risk score (≥4): PPV 65.2% (42.7–83.6%) C statistic 0.879 in the derivation and 0.857 in the validation cohort	*Strengths:* Standardized score Large sample size, two-center study *Limitations:* Combination with arterial thrombosis No external validation
Bastos-Oreiro, 2021, Retrospective [16]	208	NHL, HL	TiC-LYMPHO compared to Khorana and ThroLy scores	None	Genetic risk score, type of lymphoma, mediastinal involvement, Ann Arbor stage, bed rest for >3 days, family or personal history of VTE, BMI > 25	31 (14.9%), follow-up 6 months EPV: ~4	No patient received thromboprophylaxis at baseline. No information on thrombophilia screening.	Sensitivity TiC-LYMPHO vs. Khorana vs. ThroLy: 93.5 vs. 6.45 vs. 19.35% Specificity TiC-LYMPHO vs. Khorana vs. ThroLy: 54.5 vs. 94.0 vs. 96.4% PPV TiC-LYMPHO vs. Khorana vs. ThroLy: 26.3 vs. 16.6 vs. 50% NPV TiC-LYMPHO vs. Khorana vs. ThroLy: 97.9 vs. 84.4 vs. 86.6% AUC TiC-LYMPHO vs. Khorana vs. ThroLy: 0.783 vs. 0.502 vs. 0.579	*Strengths:* Innovative model Discrimination statistics *Limitations:* Small sample size Expensive genetic testing No external validation No optimism correction
Dharmavaram 2020, Retrospective [17]	790	DLBCL, FL	Lymphoma-specific venous thrombosis prediction model	Internal	Khorana score vs. lymphoma-significant variables such as lymphoma subtype, albumin, WBC count and bulky disease	106 VTE (13.4%), follow-up 49 months: 21 PE 85 DVT DLBCL 5-year cumulative incidence: 16.3% FL 5-year cumulative incidence: 3.8% EPV: ~11	No patient received thromboprophylaxis at baseline. No information on thrombophilia screening.	Khorana score at 2 years: Sensitivity 60%, specificity 63%, C-index 0.601 Proposed model at 2 years: Sensitivity 82%, specificity 68%, C-index 0.775	*Strengths:* Relevant, easily accessible predictors Discrimination statistics *Limitations:* Single center, long enrolment time No external validation
Hantrakun, 2024, Retrospective [18]	591	DLBCL	Age-adjusted IPI	N/A	Ann Arbor stage III/IV, serum LDH > normal, and ECOG performance status 2–4 (in patients ≤ 60 years)	32 VTE, follow-up 1 year (one-year cumulative incidence of VTE of 5.4%): 16 DVT 8 isolated PE 2 DVT with PE 6 other venous thrombosis EPV: ~8	No patient received thromboprophylaxis at baseline. No information on thrombophilia screening.	Estimated 1-year cumulative incidence of VTE: 3.0% in age-adjusted IPI < 2 (low to low–intermediate risk) vs. 9.7% in age-adjusted IPI ≥ 2 (high–intermediate to high risk) (HR, 3.5; 95% CI 1.6–7.8) C-statistic of age-adjusted IPI was 0.65 (95% CI, 0.58–0.72)	*Strengths:* Validated prognostic index Competing risk analysis Discrimination statistics *Limitations:* Single center Wide confidence intervals Low event rate
He, 2025, Retrospective [19]	605	NHL	Simp-SMOTE_rf_GBM	Internal	Anticoagulation, D-dimer, LDH, venous catheterization, CEA, ECOG score, total proteins, total cholesterol, infectious diseases, β2- microglobulin, calcium, ESAs/G-CSFs, hemoglobin, mediastinal involvement, central involvement	61 VTE (10.08%) within 6 months after diagnosis: 41 DVT 5 isolated PE 15 other venous thrombosis EPV: ~4	36.5% of patients received prior anticoagulation. No information on thrombophilia screening.	AUC 0.954 (95% CI: 0.932–0.976), Sensitivity 89%, Specificity 88%, NPV 64.7% and PPV 97%	*Strengths:* Discrimination statistics *Limitations:* Single center Only hospitalized patients Anticoagulant use classified as predictor No external validation Very high AUC
Jiang, 2025, Retrospective [27]	1141	HL, DLBCL, TCL, NK/TCL	VTE-EWS (early warning system) vs. Khorana score	External	WBC, D-dimers, central venous catheter use, age, chemotherapy cycles, ECOG score, a predicted probability > 0.7 implies a high risk for VTE	124 VTE (10.86%) EPV: ~6	No information on prior thromboprophylaxis or thrombophilia screening	Specificity in low-risk patients: 91% for VTE-EWS vs. 77% for Khorana score Sensitivity in high-risk patients: 65% for VTE-EWS vs. 54% for Khorana score	*Strengths:* External validation Comparison with validated tools Multicenter study *Limitations:* Only hospitalized patients No calibration assessment Classification metrics threshold-dependent, leading to low sensitivity
Li, 2024, Retrospective [20]	325	NHL	Khorana score, ThroLy, modified ThroLy	None	See reference [29]. Additional improvement of the ThroLy score by adding the level of D-dimer ≥ 1345 μg/dL and adjusting hemoglobin to <110 g/L	21 VTE (6.4%), median time 2 months after diagnosis: 9 DVT upper limb 6 DVT lower limb 4 subclavian and jugular DVT 2 left head vein thrombosis EPV: ~3–4	No patient received thromboprophylaxis at baseline. No information on thrombophilia screening.	Khorana score Sensitivity: 14.3% Specificity: 91.8% AUC: 0.639 ThroLy score Sensitivity: 31.8% Specificity: 89.8% AUC: 0.695 Modified ThroLy score Sensitivity: 76.2% Specificity: 71.4% AUC: 0.738	*Strengths:* Identification of potential improvements to an already existing tool *Limitations:* Single center Not routinely available predictors Cutoff optimization is driven by the same dataset
Liang, 2023, Prospective [21]	1069	All types of lymphoma	Nomogram model for predicting VTE risk	Internal	Age, gender, platelet count, D-dimer and chemotherapy cycle	52 (4.92%) VTE, median time 3.4 months EPV: ~10	No information on prior thromboprophylaxis or thrombophilia screening	AUC at 1 year: 0.838 Royston D statistics of 1000 cross-validations: 1.61 ± 0.07, indicating very good discrimination power	*Strengths:* Routinely available predictors Robust internal validation Good discrimination statistic (Royston D statistic) Time-to-event analysis *Limitations:* Single center No external validation
Ma, 2024, Retrospective [31]	13,025	All types	VTE RAM	External, two validation cohorts	Histological subtype, pretreatment BMI, type of treatment, hospitalization length, previous VTE, immobilization, time span to treatment beginning	VTE incidence: Derivation cohort (10,313 patients): 5.8% First validation cohort (854 patients): 8.2% Second validation cohort (1858 patients): 8.8% EPV: ~74	No patient received thromboprophylaxis at baseline. No information on thrombophilia screening.	*Derivation cohort:* C-statistic for overall VTE: 0.68 (95% CI 0.67–0.69) C-statistic for PE/low extremities-DVT: 0.68 (95% CI, 0.66–0.71) *First validation cohort:* C-statistic for overall VTE: 0.69 (95% CI 0.64–0.79) C-statistic for PE/low extremities-DVT: 0.72 (95% CI, 0.65–0.79) *Second validation cohort:* C-statistic for overall VTE: 0.72 (95% CI 0.65–0.79) C-statistic for PE/low extremities-DVT: 0.69 (95% CI, 0.63–0.73)	*Strengths:* Multicenter study with large sample Routinely available predictors, selected with LASSO regression High number of events per variable External validation Good discrimination statistics Competing risk analysis *Limitations:* Retrospective design
Ma’koseh, 2024, Retrospective [22]	321	HL	Khorana, ThroLy	N/A	See references [11,29]	15 (4.7%) with a median follow-up of 6.9 (0.3–42.1) months: 12 upper extremities 2 PE 1 lower extremity EPV: ~3	No information on prior thromboprophylaxis or thrombophilia screening	No correlation between VTE and Khorana score > 2 (*p* = 0.689) or ThroLy score > 3 (*p* = 0.335) Older age (*p* = 0.014) and relapsed or refractory disease (*p* = 0.003) significantly correlated with VTE	*Strengths:* Validated RAMs and predictors *Limitations:* Single center Focus only on HL No discrimination statistics
Pan, 2025, Retrospective [23]	790	NHL and HL	Lymphoma-specific nomogram, patients split 7:3 into development and internal-validation cohorts	Internal	ECOG score, coronary heart disease, prior VTE, central venous catheterization, D-dimer	77 thrombotic events (9.8%): 50 upper extremities 14 lower extremities 10 intracranial thromboses 2 arterial thromboses 1 PE EPV: ~11	No information on prior thromboprophylaxis. No thrombophilia screening.	*AUC in the development cohort:* 0.5 years: 0.813 1 year: 0.818 2 years: 0.733 *AUC in the validation cohort:* 0.5 years: 0.724 1 year: 0.731 2 years: 0.659 AUC of ThroLy at 1 year: 0.587 AUC for Khorana at 1 year: 0.527	*Strengths:* Validated RAMs and predictors Calibration plots *Limitations:* Single center No external validation
Rupa-Matysek 2018, Retrospective [24]	428	DLBCL, HL	Khorana score and other predictors	N/A	See reference [11]	64 (15%) in the median follow-up period of 4.7 months (1.4–7.6): 18 lower extremities 7 PE 39 DVT (23 internal jugular veins, 1 portal vein, 15 upper extremities) EPV: ~12	No patient received thromboprophylaxis at baseline. No information on thrombophilia screening.	Khorana score did not adequately predict VTE (PPV 15%, NPV 82%, C-statistic 0.51) *Risk factors associated with VTE:* Bulky disease (OR 2.34; 1.62–3.36) Poor prognostic disease (OR 1.32; 1.01–1.74) DLBCL (OR 1.61; 1.17–2.19)	*Strengths:* Validated RAMs and predictors Discrimination statistics High number of events per variable *Limitations:* Single center No calibration assessment No evaluation of the risk factors in form of a prediction model
Rupa-Matysek 2018, Retrospective [25]	428	DLBCL, HL	ThroLy	N/A	See reference [29]	64 (15%) in the median follow-up period of 4.7 months (1.4–7.6): 18 lower extremities 7 PE 39 DVT (23 internal jugular veins, 1 portal vein, 15 upper extremities) EPV: ~12	No patient received thromboprophylaxis at baseline. No information on thrombophilia screening.	48% of the VTE events occurred in the low-risk ThroLy score group: C-statistic 0.55 (AUC 95% CI: 0.40–0.70)	*Strengths:* Validated RAMs and predictors *Limitations:* Single center No calibration assessment Wide confidence intervals No report of sensitivity, specificity, PPV and NPV
Santi, 2017, Retrospective [30]	1717	DLBCL, INHL, MCL, FL	Khorana score	N/A	See reference [11]	53 VTE events The six-month incidence of severe VTE (CTCAE grade ≥ 3) was 0.7%, rising to 3% for any grade of VTE EPV: ~10	Thromboprophylaxis used in two studies included. No information on thrombophilia screening.	Khorana score categories were positively associated with the risk of VTE of any grade (Gray’s test *p*-value = 0.048) and with severe events (grade ≥ 3; Gray’s test *p*-value = 0.012) A higher Khorana score is associated with a higher incidence of any grade of VTE event VTE incidence is higher in patients with DLBCL (HR: 3.42, 95% CI: 1.32–8.84)	*Strengths:* Validated RAM Multicenter study, large sample size Competing risk analysis *Limitations:* Low number of events per variable No discrimination statistics Missing data
Wang, 2024, Retrospective [28]	305	Not mentioned	Nomogram for detecting PICC-associated thrombosis	External	Activity amount, thrombosis history in the last 12 months, ATIII, total cholesterol and D-dimer	35 (11.5%) PICC-related VTE, median time 13 days EPV: ~7	No patient received thromboprophylaxis at baseline. No information on thrombophilia screening.	AUC in the training set: 0.907, 95%CI: 0.850–0.964 AUC in the validation set: 0.896, 95%CI: 0.782–1.000	*Strengths:* Multicenter study Routinely available predictors External validation *Limitations:* Focus exclusively on PICC-related thrombosis Wide confidence interval in the validation cohort Low event rate per variable with a very high AUC.
Yang, 2021, Retrospective [26]	555	All types	Nomogram based on prognostic factors for predicting VTE	Internal	Platelet count, hemoglobin level, gender, clinical stage, type of lymphoma (HL vs. B cell)	113 VTE events (20.3%): 42 DVT of upper extremities 71 DVT of lower extremities EPV: ~22	No patient received thromboprophylaxis at baseline. No information on thrombophilia screening.	AUC of the nomogram: 0.731, 95%CI: 0.682–0.781, C-index 0.73 AUC of Khorana score: 0.557, 95%CI: 0.495–0.61	*Strengths:* All types of lymphoma are included Calibration curve Comparison with Khorana score High number of events per variable *Limitations:* Single center No external validation

AMI = acute myocardial infarction, ATIII = antithrombin III, AUC = area under the curve, BMI = body mass index, CEA = carcinoembryonic antigen, CI = confidence interval, CLL/SLL = chronic lymphocytic leukemia/small lymphocytic lymphoma, CTCAE = Common Terminology Criteria for Adverse Events, DLBCL = diffuse large B-cell lymphoma, DVT = deep vein thrombosis, ECOG = Eastern Cooperative Oncology Group Performance Status, EPV = events per variable, ESAs/G-CSFs = erythropoiesis stimulating agents/granulocyte colony-stimulating factors, FL = follicular lymphoma, HL = Hodgkin lymphoma, HR = hazard ratio, INHL = indolent non-Hodgkin lymphoma, IPI = International Prognostic Index, LASSO = Least Absolute Shrinkage and Selection Operator, LDH = lactate dehydrogenase, MCL = mantle cell lymphoma, N/A = not applicable, NHL = non-Hodgkin lymphoma, NK/TCL = natural killer/T-cell lymphoma, NPV = negative predictive value, OR = odds ratio, PE = pulmonary embolism, PICC = peripherally inserted central catheter, PPV = positive predictive value, RAM = risk assessment model, TCL = T-cell lymphoma, VTE = venous thromboembolism, WBC = white blood cells. Italics is used to differentiate between groups of data.

**Table 3:** Frequent predictors for VTE evaluated by multivariate analysis.

Predictor	Study/Type of Validation/Concerns Regarding Applicability			
	Authors	Validation	Results	Concerns
Previous VTE	Antic [29]	Internal (sample splitting)	OR 14.1 (4.4–45), *p* < 0.001	Increased risk of overfitting
	Pan [23]	Internal (sample splitting)	HR 6.5 (2.0–21.5), *p* = 0.02	
	Bastos [16]	No validation	HR 4.1 (1.4–11.8), *p* = 0.003	
	Li [20]	No validation	OR 105.3, *p* < 0.001	
	Ma [31]	External validation	SHR 2.7 (2.2–3.5)	
	Abdel [15]	External validation for [29]	OR 1.6 (0.7–3.8), *p* = 0.23	
ECOG performance status	Antic [29]	Internal (sample splitting)	ECOG 2–4: OR 5.1 (1.9–14.0), *p* < 0.001	Increased risk of overfitting
	Pan [23]	Internal (sample splitting)	ECOG ≥ 4: HR 6.0 (1.8–19.4), *p* = 0.003	
	Li [20]	No validation	ECOG ≥ 3: OR 2.9, *p* = 0.13	
	Abdel [15]	External validation for [29]	ECOG 2–4: OR 1.8 (0.9–3.6), *p* = 0.054	
BMI	Antic [29]	Internal (sample splitting)	BMI ≥ 30: OR 10.7 (3.3–34.6), *p* < 0.001	Increased risk of overfitting
	Ma [31]	External validation	BMI ≥ 35: SHR 1.3 (1.1–1.6)	
DLBCL	Rupa-Matysek [25]	External validation for [29]	OR 1.9 (1.05–3.4), *p* = 0.034	
	Rupa-Matysek [24]	External validation for [10]	OR 1.6 (1.1–2.1), *p* = 0.003	
CVC	Pan [23]	Internal (sample splitting)	HR 2.4 (1.1~5.0), *p* = 0.01	Increased risk of overfitting
Mediastinal involvement	Antic [29]	Internal (sample splitting)	OR 8.0 (4.0–15.8), *p* = 0.001	Increased risk of overfitting
	Li [20]	No validation	OR 11.2, *p* = 0.001	
Hemoglobin <100 g/L	Antic [29]	Internal (sample splitting)	OR 3.9 (1.7–8.5), *p* = 0.001	Increased risk of overfitting
	Abdel [15]	External validation for [29]	OR 2.7 (1.4–5.4), *p* = 0.003	

BMI = body mass index, CVC = central venous catheter, DLBCL = diffuse large B-cell lymphoma, ECOG = Eastern Cooperative Oncology Group Performance Status, HR = hazard ratio, OR = odds ratio, VTE = venous thromboembolism.

**Table 4:** Characteristics of the studies assessing predictors of VTE.

Authors, Year, Type of Study	Number of Patients	Type of Lymphoma	Studied Predictors	VTE Incidence	Prophylactic Anticoagulation	Results
Borchmann, 2019, Prospective [32]	5773	HL	Type of treatment, age, gender, smoking, platelet count, anemia, leukocyte count, BMI, Khorana score, B-symptoms, albumin, ECOG, extranodal disease, mediastinal mass, smoking, erythropoietin	3.3% incidence of thrombosis, 175 venous thromboses 81 DVT in the arm 43 DVT of the leg 23 PE 28 other thromboses 30.6% CVC-associated	2 events occurred despite thromboprophylaxis.	Higher incidence of thrombosis based on the treatment received: 9.4% in 8xBEACOPP-14 vs. 5.7% in 8xBEACOPPesc (OR 1.74, *p* = 0.007) Age and smoking were associated with thrombosis. A higher Khorana score did not increase venous thrombotic risk (OR (per point) = 0.92, *p* = 0.41)
Borg, 2016, Retrospective [33]	289	DLBCL	Overweight, smoking, thromboprophylaxis, history of VTE, Ann Arbor stage III-IV, ECOG, B-symptoms, IPI score, albumin, platelet count, hemoglobin, leukocyte count, LDH	32 events (11%), follow-up 16 months 19 DVT 13 PE	100 patients with thromboprophylaxis, 9 developed a thrombotic event.	*Predictors of VTE:* Past history of VTE, HR 4.3 (1.3–14.1) Ann Arbor stage III–IV, HR 2.8 (1.1–6.8) ECOG 3–4, HR 2.8 (1.0–7.2) IPI score: high risk, HR 3.9 (1.2–12.7) The risk of VTE was higher before chemotherapy was initiated (IR 0.6 before treatment, 0.18 during treatment, and 0.04 after treatment)
Byun, 2019, Retrospective [34]	235	PCNSL	Age, gender, radiation, type and dose of chemotherapeutic agents, body mass index, number of brain parenchymal lesions, platelet count and leukocyte count, ECOG, hemoglobin	33 events (14%), follow-up 21 months 11 DVT 15 PE 7 DVT with PE	Not mentioned.	*Predictors of VTE:* Female gender, HR 2.3 (1.1–4.9) Age > 60 years, HR 3.2 (1.3–8.0) ECOG performance ≥ 2, HR 3.6 (1.4–9.4) Hemoglobin < 10 g/dL (0.8–11.8)
Chen, 2022, Retrospective [35]	1069	HL, DLBCL, TCL, NK/T-cell lymphoma	Age, gender, body mass index, ECOG, Ann Arbor stage, CVC, leukocyte and platelet counts, hemoglobin, chemotherapy regimen and number of cycles, D-dimer	52 events (4.9%), follow-up 23 months	Not mentioned.	*Predictors of VTE:* Male sex, HR 2.2 (1.1–4.3, *p* = 0.012) Age > 64 years, HR 2.2 (1.0–5.0, *p* = 0.045) Number of cycles of chemotherapy 1–5, HR 4.5 (1.1–17.8, *p* = 0.029) Platelet count ≥ 350 × 109/L, HR 2.5 (1.1–5.4, *p* = 0.016) D-dimer > 0.5 mg/L, HR 4.3 (2.1–8.9, *p* < 0.001)
El-Ashwah, 2024, Retrospective [2]	777	NHL	ECOG, laboratory parameters, bulky lesions, liver cirrhosis, IPI, treatment response and relapse status	107 VTE events (13.7%)	Not mentioned.	*Predictors of VTE at diagnosis:* ECOG ≥ 2, OR 2.1 (1.1–4.1, *p*= 0.02) Bulky lesions, OR 2.7 (1.4–5.2, *p* = 0.002) Mediastinal masses, OR 5.0 (2.1–12.2, *p* < 0.001) *Predictors of VTE in chemotherapy*: ECOG ≥ 2, HR 6.1 (1.8–19.5, *p* = 0.003) ANC, HR 1.2 (1.0–1.5, *p* = 0.03) NLR, HR 1.1 (1.1–1.3, *p* = 0.04)
Gangaraju, 2019, Retrospective [36]	734	NHL	BMT	58 VTE events (7.9%) after bone marrow transplant, median follow-up 8.1 years Cumulative VTE incidence after allogenic BMT: 14.9 ± 2.6% at 10 years Cumulative VTE incidence after autologous BMT: 5.4 ± 1.1% at 10 years	Not mentioned.	*Predictors of VTE among allogenic BMT survivors:* BMI 25–30, HR 3.5 (1.4–8.6), *p* = 0.006; History of chronic GvHD, HR 3.3 (1.5–6.9), *p* = 0.001 *Predictors of VTE among autologous BMT survivors:* Coronary artery disease, HR 5.9 (1.7–20.7), *p* = 0.005 Treatment with carmustine, HR 4.9 (1.6–14.5), *p* = 0.004
Gangaraju, 2022, Retrospective [37]	5537	DLBCL	Age > 80, history of VTE, gender, race, prior anticoagulation	524 VTE events (9.5%), median follow-up 12 months	639 (11.5%) patients taking anticoagulants prior to lymphoma diagnosis.	*Predictors of VTE:* Pre-cancer VTE history, HR 5.3 (4.3–6.6) *Protective factors:* Asian individuals, HR 0.5 (0.2–1.0) Atrial fibrillation, HR 0.3 (0.2 to 0.6)
Hashmi, 2020, Retrospective [38]	148	LBCL	Predictors of VTE after CAR T-cell therapy	16 VTE events (11%) in the first 100 days after CAR T-cell therapy	12 patients taking anticoagulants prior to CAR T-cell therapy due to previous VTE.	Bulky disease > 10 cm, bridging therapy and ECOG 2–4 were associated with a new VTE event after CAR T-cell therapy (*p* < 0.01)
Hohaus, 2018, Retrospective [39]	857	DLBCL, HL, FL, PTCL, MCL	Age, gender, histology, bulky disease, stage, ECOG, leukocyte and platelet count, hemoglobin, albumin, LDH in patients requiring hospitalization	95 VTE events (11.1%), median follow-up 14 months	Not mentioned.	*Predictors for VTE:* PCNSL, incidence of 27.2% (9/33), OR 3.7 (1.4–9.6), *p* = 0.008 Bulky disease > 10 cm, OR 3.2 (1.8–5.6), *p* = 0.0001 ECOG 2–4, OR 1.8 (1.0–3.1), *p* = 0.04 Higher incidence in aggressive lymphomas (DLBCL 12.6%, PTCL 13.1%) vs. HL (6.8%) or FL (5%)
Kirkizlar, 2020, Retrospective [4]	150	HL	Age, gender, histology, stage, ECOG, anemia, leukocyte and platelet count, BMI, thromboprophylaxis	31 VTE events (20.7%): 18 upper extremity DVT 3 lower extremity DVT 10 PE	17 patients receiving thromboprophylaxis.	VTE timing, CVC, initial high fibrinogen level, initial leukocytosis, and prior thromboprophylaxis were associated with VTE
Lan, 2021, Retrospective [40]	668	TCL	Age, gender, stage, ECOG, B-symptoms, CVC, hemoglobin, leukocyte and platelet count, LDH, albumin, D-dimer	33 VTE events (4.9%), all DVT	Not mentioned.	*Predictors for VTE:* CVC, OR 3.2 (1.4–7.2), *p* = 0.003 Stage III–IV, OR 2.3 (1.0–4.9), *p* = 0.035
Lekovic, 2010, Retrospective [41]	42	PMBCL	Age, gender, tumor mass, superior vena cava syndrome, therapy response	15 VTE events (35.7%): 14 DVT 1 PE	Thrombophilia diagnostic performed in 11 patients with VTE 1 patient with prior VTE history developed VTE, no patient without VTE had prior anticoagulation.	*Predictors for VTE:* Higher fibrinogen level (8.2 ± 2.6 g/L vs. 6.3 ± 2.2 g/L, *p* = 0.02) D-dimer level (509 ± 276.8 μg/L vs. 207 ± 166.7 μg/L, *p* = 0.001) Larger diameter of mediastinal tumor mass (14 ± 3.1 cm vs. 11 ± 3.4, *p* = 0.01)
Lim, 2015, Prospective [42]	322	DLBCL	Age, gender, ECOG, smoking, BMI, stage, hemoglobin, leukocyte and platelet count, LDH	34 VTE events (10.6%), follow-up 41 months: 18 DVT 12 PE 3 PE with DVT	No patients received thromboprophylaxis.	*Predictors for VTE:* Age > 60 years, SHR 2.6 (1.5–4.5), *p* < 0.01 ECOG 2–4, SHR 2.0 (1.0–3.7), *p* = 0.03
Lund, 2015, Retrospective [43]	10,924	All types of lymphoma	Transient effect of chemotherapy, radiation, CVC, rituximab	355 VTE events (3%), follow-up 2 years 115 PE 195 DVT 45 other VTE	Not mentioned.	*Predictors for VTE:* Central nervous system involvement, SHR 2.5 (1.5–4.1) PTCL with SHR 2.0 (1.2–3.3), DLBCL with SHR 1.7 (1.3–2.4) and HL with SHR 1.6 (1.0–2.4) vs. indolent lymphoma CVC use increased VTE risk, aOR 6.7 (1.2–28.1)
Mahajan, 2020, Retrospective [44]	992	PCNSL	Age, gender, chemotherapy, prior VTE	143 VTE events (14.4%), follow-up 58 months: 75 PE 32 proximal DVT 36 distal DVT	Not mentioned.	*Predictors of VTE:* Age 50–59, HR 2.5 (1.1–6.0) Initial course of chemotherapy, HR 2.4 (1.3–4.4) and radiation, HR 1.5 (1.0–2.2) *Protective factors for VTE:* Asian/Pacific Islanders, HR 0.3 (0.2–0.6) vs. non-Hispanic Whites Prior VTE history, HR 1.4 (0.4–4.7)
Ma’koseh, 2023, Retrospective [45]	216	Relapsed DLBCL, HL	Histology, mediastinal involvement, BMI, LDH, ThroLy, hospital stay	36 VTE events (16.7%): 28 upper extremity DVT 4 lower extremity DVT 3 PE	One patient who developed VTE had previous thromboprophylaxis.	*Predictors for VTE:* High LDH level, OR 6.5 (2.5–16.7), *p* < 0.001 Mediastinal involvement, OR 2.7 (1.2–5.6), *p* = 0.005 Hospital stay ≥ 24 days, OR 2.7 (1.2–5.6), *p* = 0.007)
Nguyen, 2025, Prospective [46]	157	HL, NHL	Age, gender, ECOG, D-dimer, cardiovascular disease, bulky disease	13 VTE events	Patients with Khorana score ≥ 3 (7.6%) received prophylactic anticoagulation.	*Predictors for VTE:* D-Dimer (≤500 vs. >500 ng/mL), OR 0.04 (0.003–0.6), *p* = 0.02 Cardiovascular comorbidities (Yes vs. No), OR 0.03 (0.002–0.5), *p* = 0.01
Otasevic, 2022, Prospective [6]	706	HL, NHL	Erythrocyte sedimentation rate, C-reactive protein, Neutrophil–Lymphocyte Ratio, Platelet–Lymphocyte Ratio, LDH, total protein, albumin	69 VTE events (9.8%), median follow-up 25 months: 39 DVT of the extremities 16 PE 26 other sites for DVT	Almost 70% of patients received thromboprophylaxis in the last three years of the study.	*Predictors for VTE:* Neutrophil–Lymphocyte Ratio, OR 1.04 (1.0–1.08), *p* = 0.04 C-reactive protein, OR 1.007 (1.0–1.01), *p* = 0.024
Park, 2012, Prospective [47]	686	HL, NHL	Age, gender, ECOG, serum LDH, B-symptoms, extranodal involvement, histology, comorbidities	54 VTE events, median follow-up 21 months: 33 DVT 21 PE	No patients received thromboprophylaxis.	*Predictors for VTE:* Age > 60 years, OR 2.2 (1.1–5.4), *p* = 0.022 PCNSL, OR 4.1 (2.8–18.6), *p* < 0.01
Rupa-Matysek, 2017, Retrospective [48]	184	DLBCL	Mean platelet volume	39 VTE events (21.2%)	No patients received thromboprophylaxis.	*Predictors for VTE:* MPV ≤ 10th percentile (≤6.1 fl), OR 1.8 (1.0–3.1), *p* = 0.03 Salvage therapy, OR 2.4 (0.6–3.6), *p* < 0.001
Rupa-Matysek, 2018, Retrospective [49]	167	HL	Mean platelet volume	12 VTE events (7.2%)	No patients received thromboprophylaxis.	*Predictors for VTE:* MPV ≤ 25th percentile (≤6.8 fl), OR 2.2 (1.0–4.5), *p* = 0.03 Advanced stage, OR 2.0 (1.0–4.0), *p* = 0.03 Bulky disease, OR 2.2 (1.1–4.3), *p* = 0.01
Saito, 2021, Retrospective [50]	78	PCNSL	Age, gender, BMI, comorbidities, ECOG	24 VTE events (31%): 12 DVT 9 PE 5 PE with DVT	42 patients received perioperative thromboprophylaxis.	*Predictors for VTE:* Previous VTE, HR 4.2 (2.4–7.5), *p* < 0.001 Ambulatory status, HR 5.1 (1.5–17.1), *p* = 0.007 Initial hemoglobin < 10 g/dL, HR 7.7, (2.0–28.9), *p* = 0.003 History of diabetes, HR 2.4 (1.0–5.7), *p* = 0.04
Sanfilippo, 2016, Retrospective [51]	2037	DLBCL, FL	Age, gender, histological type, history of prior VTE, BMI, hemoglobin, LDH, stage (stage III/IV versus I/II), B-symptoms, treatment with doxorubicin and time period during chemotherapy administration	246 VTE events (12.1%), follow/up 28 months	Not mentioned.	*Predictors for VTE:* Previous VTE, adjusted HR 4.7 (2.4–9.0), *p* < 0.0001 During chemotherapy, adjusted HR 7.6 (4.7–12.2), *p* < 0.0001 Stage III & IV, adjusted HR 1.4 (1.0–2.1), *p* = 0.02 B-symptoms, adjusted HR 1.4 (1.0–2.0), *p* = 0.02 BMI ≥ 30, adjusted HR 1.6 (1.0–2.3), *p* = 0.02
Yokoyama, 2012, Retrospective [52]	142	DLBCL	Age, gender, BMI, ECOG, IPI score, CVC	13 VTE events: 11 DVT 2 PE with DVT	2 patients received anticoagulation prior to lymphoma diagnosis (1 developed VTE).	*Predictors for VTE:* ECOG 2–4, OR 31.1 (3.7–255.6), *p* = 0.001
Yuen, 2020, Retrospective [53]	51	PCNSL	Age, gender, ECOG, hemoglobin, leukocyte and platelet count, Khorana score	13 VTE events (25%): 5 DVT 1 PE 3 PE with DVT 4 CVC-associated	38 patients received thromboprophylaxis (10 developed VTE).	Patients with Khorana score ≥ 2 were more likely to have VTE than those with a Khorana score < 2 (60% vs. 15%; *p* = 0.01)
Zhou, 2010, Retrospective [54]	422	HL, NHL	Age, gender BMI, laboratory parameters, comorbidities	80 VTE events: 59 DVT 17 PE 4 PE with DVT	18 patients received anticoagulation prior to lymphoma diagnosis.	*Predictors for VTE:* Female gender, OR 3.5 (1.6–7.4), *p* = 0.001 High hemoglobin, OR 1.2 (1.0–4.5), *p* = 0.02 High serum creatinine, OR 3.2 (1.3–7.8), *p* = 0.009 Doxorubicin- or methotrexate-based chemotherapy, OR 3.4 (1.5–7.7), *p* = 0.003

ANC = absolute neutrophil count, BEACOPP = bleomycin, etoposide, doxorubicin, cyclophosphamide, vincristine, procarbazine, prednisone, BMI = body mass index, BMT = bone marrow transplant, CAR = chimeric antigen receptor, CVC = central venous catheter, DLBCL = diffuse large B-cell lymphoma, DVT = deep vein thrombosis, ECOG = Eastern Cooperative Oncology Group Performance Status, FL = follicular lymphoma, GvHD = graft versus host disease, HL = Hodgkin lymphoma, HR = hazard ratio, IPI = International Prognostic Index, LBCL = large B-cell lymphoma, LDH = lactate dehydrogenase, MCL = mantle cell lymphoma, MPV = mean platelet volume, NHL = non-Hodgkin lymphoma, NK/TCL = natural killer/T-cell lymphoma, (a) OR = (adjusted) odds ratio, PCNSL = primary central nervous system lymphoma, PE = pulmonary embolism, PMBCL = primary mediastinal B-cell lymphoma, SHR = subdistribution hazard ratio, TCL/PTCL = peripheral T-cell lymphoma, VTE = venous thromboembolism. Italics is used to differentiate between groups of data.

## Data Availability

No new data were created in this study. Data sharing is not applicable to this article.

## Supplementary Materials

The following supporting information can be downloaded at: https://www.mdpi.com/article/10.3390/curroncol33070401/s1, Supplementary File S1: Search strategy; Supplementary File S2: PRISMA checklist.

## Abbreviations

The following abbreviations are used in this manuscript:

AMI	acute myocardial infarction
ANC	absolute neutrophil count
ATIII	antithrombin III
AUC	area under the curve
BEACOPP	bleomycin, etoposide, doxorubicin, cyclophosphamide, vincristine, procarbazine, prednisone
BMI	body mass index
BMT	bone marrow transplant
CAR	chimeric antigen receptor
CVC	central venous catheter
CEA	carcinoembryonic antigen
CI	confidence interval
CLL/SLL	chronic lymphocytic leukemia/small lymphocytic lymphoma
CTCAE	Common Terminology Criteria for Adverse Events
DLBCL/LBCL	diffuse large B-cell lymphoma/large B-cell lymphoma
DVT	deep vein thrombosis
ECOG	Eastern Cooperative Oncology Group Performance Status
EPV	events per variable
ESAs/G-CSFs	erythropoiesis stimulating agents/granulocyte colony-stimulating factors
FL	follicular lymphoma
GvHD	graft versus host disease
HL	Hodgkin lymphoma
HR/SHR/aHR	hazard ratio/subdistribution hazard ratio/adjusted hazard ratio
IL	interleukin
INHL	indolent non-Hodgkin lymphoma
IPI	International Prognostic Index
LASSO	Least Absolute Shrinkage and Selection Operator
LDH	lactate dehydrogenase
MCL	mantle cell lymphoma
MPV	mean platelet volume
N/A	not applicable
NET	neutrophil extracellular trap
NHL	non-Hodgkin lymphoma
NK/TCL	natural killer/T-cell lymphoma
NPV	negative predictive value
OR/aOR	odds ratio/adjusted odds ratio
PCNSL	primary central nervous system lymphoma
PE	pulmonary embolism
PICC	peripherally inserted central catheter
PMBCL	primary mediastinal B-cell lymphoma
PPV	positive predictive value
PRISMA	Preferred Reporting Items for Systematic Reviews and Meta-analysis
PROBAST	Prediction model Risk of Bias Assessment Tool
PROSPERO	International Prospective Register for Systematic Reviews and Meta-analysis
RAM	risk assessment model
TCL/PTCL	T-cell lymphoma/peripheral T-cell lymphoma
TF	tissue factor
ThroLy	Thrombosis Lymphoma predictive score
TNF-α	tumor necrosis factor-α
VTE	venous thromboembolism
WBC	white blood cells
