# Supplementary material for: Predictors and Risk Assessment Models for Venous Thromboembolism in Patients Diagnosed with Lymphoma: A Systematic Review
Source: Curr Oncol. 2026 Jul 4;33(7):401. doi: 10.3390/curroncol33070401 (PMC13408612; doi:10.3390/curroncol33070401)
Supplement: Supplementary file 1 [file curroncol-33-00401-s001.zip › Supplementary File S1.pdf]

Supplementary file S1. Search strategy

Pubmed: 329 results

(("venous thromboembolism"[MeSH Terms] OR "pulmonary embolism"[MeSH Terms] OR "thrombosis"[MeSH Terms]) AND "lymphoma"[MeSH Terms]) AND ((humans[Filter]) AND (english[Filter]) AND (alladult[Filter]) AND (1975:2026[pdat]))

Scopus: 104 results

('venous thromboembolism':ti OR 'vein thrombosis':ti OR 'lung embolism':ti) AND 'lymphoma':ti AND [1975-2026]/py

Embase: 159 results

(TITLE (lymphoma) AND TITLE (venous thromboembolism) OR TITLE (pulmonary embolism) OR TITLE (thrombosis)) AND (LIMIT-TO (LANGUAGE , "English")) AND (LIMIT-TO (EXACTKEYWORD , "Human") OR LIMIT-TO (EXACTKEYWORD , "Adult") OR LIMIT-TO (EXACTKEYWORD , "Humans")) AND (LIMIT-TO (SUBJAREA , "MEDI")) AND (LIMIT-TO (DOCTYPE , "ar")) AND (LIMIT-TO (SRCTYPE , "j")) AND (LIMIT-TO (PUBSTAGE , "final"))
